# Garden Cities for Consumptives: Administrative Problems

**Published:** 1913-11-29

**Authors:** J. E. Esslemont


### GARDEN CITIES FOR CONSUMPTIVES.*

### III.—Administrative Problems.

By J. E. ESSLEMONT, M.B.

In discussing this scheme I have frequently been asked, "But wouldn't it be very dangerous to have such a number of infectious cases together?" There seems to be a vague notion that infectious cases, like an army, would be more formidable when massed together than when scattered abroad. It needs very little reflection, however, to show that the exact opposite is the truth—that the risk is great just in proportion as infectious cases are scattered among the unaffected population, in the same way that seeds grow and multiply when scattered in suitable soil, but not when they are kept in a packet.

### Infection in the Colony.

"But the colonies themselves must surely be hot-beds of infection, and healthy people or children who enter them are almost bound to contract the disease." Even that I do not admit. Surgeons and hospital nurses are constantly handling much more infectious diseases than tuberculosis, but rarely get infected. Risks of infection are great and very difficult to avoid in the general community under present conditions, but in the colonies the conditions would be entirely different. Segregation would be practised within the colony as well as without. The most advanced and dangerous cases would all be in hospital. In the case of the others strict regulations would be enforced, as in well-conducted sanatoria, for the proper disposal of sputum, the prompt burning or disinfection of handkerchiefs, the scrupulous cleanliness of person and of rooms, and the other precautions necessary to prevent infection and reinfection. In colonies where every inhabitant was trained in proper hygienic habits, where buildings, furniture, and everything else were designed for the purpose, and where Nature's disinfectants—sunshine and fresh air—were freely admitted everywhere, it would be possible to reduce the risks of infection almost to vanishing point in a way that could never be done in the general community.

Notwithstanding the presence of infectious cases the colonies would still be the best places for the cure of consumption, as good sanatoria are to-day. Having eradicated tuberculosis from the general community, unrelenting war would be waged on it in its last stronghold, and all the resources of science brought to bear on its extinction. A fresh infection could only occur through negligence of proper precautions, and any such case would be the occasion of strict inquiry and of redoubled precautions in future. The disease would gradually be confined to narrower and narrower circles within the colonies, until, by the cure of all curable cases and the death of the incurable, the nation would at last be freed from the scourge which has decimated it for centuries.

As previously mentioned, I consider that when no better arrangements can be made, the children of a tuberculous parent might be allowed to enter the colony. As shown in the last paragraph, the risks would be very small. The infected member of the family would be separated from the others to the extent necessary for their safety. The children would live a healthy open-air life which would render them comparatively immune, and they would grow up under the eyes of skilled medical men, so that if the slightest sign of disease appeared it would be met by prompt and efficient treatment.

In many cases careful examination and testing would probably reveal the fact that the children of tuberculous parents were already infected, in which case the colony would undoubtedly be the best place for them.

### The Question of Compulsion.

Any attempt to make segregation of all infectious cases compulsory at the present time is out of the question owing to the lack of suitable accommodation for the immense number of cases to be dealt with. At present the demand for sanatoria among the working classes far exceeds the supply, and if a colony scheme were started, I feel sure that the available accommodation would be eagerly sought after. The ideal to be aimed at in a segregation scheme is an arrangement which, while giving complete safety to the public, will, at the same time, give the best chance of recovery and the fullest life possible to the patient. If our scheme in its actual working approaches that ideal there will never be any need for compulsion in order to carry it out, at least so far as reasonable people are concerned. Compulsory powers should certainly be provided, but the arm of the law will only have to be invoked in the case of people who are blind to their own best interests as well as to the common weal. Once a really satisfactory method of dealing with tuberculosis is adopted, and its benefits have been demonstrated, public opinion will not only sanction, but will demand, the carrying out of preventive measures on a proper scale, and with proper stringency—just as it does in the case of smallpox or cholera at the present day.

Of course, when a patient in the colony arrived at a stage when the disease was completely arrested and he was undoubtedly non-infectious, he would be free to leave the colony. In many cases, however, he would be averse to leaving the favourable conditions under which he became cured, and arrangements should, if possible, be made whereby he might either remain on, or go to another colony provided for such cases.

Many medical men and social reformers consider other methods of dealing with tuberculosis preferable to that of segregation. Some of these methods may be briefly reviewed.

First comes *general sanitation* and *social amelioration*. There can be no doubt that these have

* Previous articles appeared on November 1 and 15.

done much and may do much more to help the campaign against tuberculosis. Better housing for the working classes, the abolition of slums and overcrowding, the advent of garden cities and town planning, better wages and shorter hours, the reform of unhealthy occupations, more temperance and less vice, hygienic education and physical training—all are important and help to diminish the incidence not only of tuberculosis, but of all other diseases as well. Even a vast improvement in the social condition of the masses, however, will not confer immunity, as is evident from the fact that consumption is by no means confined to the poor, but claims a heavy toll of victims even among the well-to-do and those whose hygienic standard of living is far higher than we can hope to secure for the general mass of the population. But we must also remember that bad, even very bad, social conditions cannot cause tuberculosis without the presence of the specific contagion.

### Incidence and Immunity.

As pointed out by Powell and Hartley (in "Diseases of the Lungs and Pleuræ," fifth edition, page 382. London: Lewis), "Persons may live in overcrowded and ill-ventilated houses for years with only generally lowered vitality, but if a case of tuberculosis supervenes the surrounding dirt and dust becomes specifically infected and tuberculosis spreads in such houses." It is doubtful, as Professor Karl Pearson has shown ("The Fight against Tuberculosis and the Death Rate from Phthisis." London: Dulau, 1911), to what extent the fall in the phthisis death-rate during the last fifty years is due to sanitary and social improvements. Epidemic diseases after a period of maximum incidence gradually become less prevalent owing to acquired immunity and the limitation of the available infectable population. There is some evidence tending to show that the English race has gradually been acquiring increased resisting power to tuberculosis, which may be partly a consequence of the prevalence of the disease itself. The English now appear to be decidedly more resistant than races such as the North American Indians, among whom the disease has recently been introduced and among whom its ravages are now far worse than in this country. It is impossible in the present state of our knowledge to gauge with any accuracy the relative importance of these factors, but it is also impossible to ascribe with any confidence the whole of the decline in the phthisis death-rate to improved sanitation. Another fact which shakes our confidence in the all-importance of general sanitation with regard to this specific disease is that since the discovery of the tubercle bacillus in 1882, and more especially since sanatorium treatment and modern hygienic methods of dealing with tuberculosis were introduced, the fall in the phthisis death-rate has been slower than during the fifteen years preceding the discovery of the bacillus.

The curve representing the fall, instead of approaching the vertical is becoming more nearly horizontal, and the date at which it seems likely to reach the zero line is receding into the indefinite future. It is clear then that all is not well with the campaign against tuberculosis. Sanitary and social reform is bound to be slow. Experience with cattle points to a method by which the disease could be wiped out in a generation—shall we hesitate to try it? Surely not. Let us get all the help we can from improved sanitation, but sanitation will be helped, not hindered, if we can find a quicker way of eradicating tuberculosis. Segregation itself is one of the most important of sanitary measures and will help the others along. Our colonies will themselves embody the latest achievements of sanitary science, and their success will give a fresh and powerful impetus to sanitary progress, so that the fears of those who think that a segregation policy for tuberculosis might divert attention from other important methods of social amelioration are groundless.

*Early diagnosis* and *curative treatment* are relied on by others as the great essentials of the campaign. If by these means we could absolutely rely on preventing cases from ever reaching the infectious stage, then, on the death of all existing infectious cases, the incidence of the disease would be stopped. Everyone knows, however, that a certain proportion of cases go steadily downhill in spite of early diagnosis and skilful treatment, and that individual cases may remain in an infectious condition for twenty or thirty years or more—carriers of the disease who may infect others with fatal results and themselves live to a green old age defying its inroads. Like sanitation, early diagnosis and curative treatment are invaluable auxiliaries but no substitute for segregation. If early treatment is better than late treatment, complete prevention is surely better still!

*Inoculation* and *tuberculin* are hailed by others as the most important weapons in the fight. The method of inoculation by live germs is, however, too uncertain and risky. Tuberculin seems to have a distinct therapeutic value in some cases, but its use has to be prolonged for months and is by no means always successful. In many cases it appears to do positive harm, and there is no evidence to show that any simple method of prophylactic inoculation with tuberculin suitable for general application, like vaccination for smallpox, is capable of conferring immunity to the disease.

### Inoculation.

Professor Metchnikoff, a great apostle of inoculation, speaking of the efforts to find an efficient tubercular vaccine, said in a recent lecture ("The Warfare against Tuberculosis," *Bedrock*, Vol. I., No. 4. January, 1913) that "after a great number of attempts in this direction the disconcerting result has been arrived at that the tubercular bacillus differs from very many other microbes in the great difficulty of conferring real immunity against its attack."

There remain the methods of *Eugenics* and *Natural Selection*. The task of evolving a race immune to tuberculosis through deliberate regulation of parentage is, however, one from which even the boldest reformer might well shrink. It would, moreover, be of doubtful benefit even if accomplished, for those who are susceptible to tuberculosis are by no means always unfit for social life in its higher forms.

On the contrary, tuberculosis has numbered among its victims many of the most honoured names in history, and has nipped in the bud countless thousands of lives of brilliant promise. One has only to mention such names as Robert Louis Stevenson, John Addington Symonds, Keats, Spinoza, Pascal, Schiller, Molière, Mozart, Schubert, Chopin, and George Sand to show that it is not only undesirables and inefficients that consumption carries off. Susceptibility to tuberculosis, *per se*, no more stamps a man as unfit than does liability to be poisoned by ptomaines or sewer gas. It only shows the need for a clean environment—free from bacterial as well as chemical poisons. If it be possible, as I believe it is, to free the country from the specific infection of tubercle, then the specific susceptibility to tuberculosis is not a matter to which the eugenic reformer need pay much heed. His concern is with future generations; ours is to see that as far as possible tuberculosis shall die out with this generation. Fortunately it is only in rare instances that children are infected before birth, so that the hope of speedy eradication is further justified on this account.

### Summary and Appeal.

It is high time that England awoke to the fact that tuberculosis is a much more active and dangerous enemy of the country than any foreign power. Foreign foes have occasionally slain their thousands, but tuberculosis is annually slaying its tens of thousands. And what, after all, is this terrible enemy of our race? A tiny microscopic organism belonging to the vegetable kingdom, which has not even the power of independent movement. Fresh air and sunshine are fatal to it. It has neither strength nor cunning, and is the most insignificant object imaginable—so long as it is outside of us. All it can do is to feed and multiply, but it is very fastidious about its food. Vegetarian or mineral diet it will have nothing to do with. Some plants eat flies, but this little parasite's normal food is living human beings—and it does not thrive so well on them when they are old and tough! This miserable parasite we foster and pamper in our midst. We give it our tender babes to feed on and poison with its excrement. We allow it to devour our young men and maidens. We even yield our men of genius, poets, philosophers, and musicians as sacrifices to its greed—or whatever corresponds to greed in a creature so insignificant.

How can we allow such things to go on? We now know the real truth about the matter, and only callousness and apathy can prevent us from taking the measures necessary to prevent this horrible slaughter of the innocents. Our attack on this parasite has hitherto been on wrong lines. We virtually ignore its existence except when it is causing obvious illness—*i.e.*, when it has firmly established itself in the vitals of a human being, where it is most difficult to kill, without killing the patient too. By the time its presence is discovered it has already done considerable damage, and our concern is then rather to repair the damage than to bother about the cause of it all. We exhaust our ingenuity and spare no expense in efforts to get the patient well. Meanwhile the invisible parasite, which has multiplied a billion-fold in the patient's lungs, and is being discharged into the outer world in millions, is allowed to scatter itself about, all unheeded, to mix with the dust of the room, to be carried about on handkerchiefs, on fingers, or on eating and drinking utensils, to settle on the furniture, to get dispersed this way and that until some of the germs accidentally get into somebody else's vitals, there to multiply unnoticed until again serious damage is done, the doctor is called in once more, and the same cycle is repeated.

### A Lesson from the Farm.

What we fail to realise is that, although it is so difficult to kill the parasite when established in the patient's tissues, it is really quite an easy thing to prevent it from finding its way from one human being to another. Put only the breadth of a field between them, and with the very simplest precautions the thing is done. Farmers find this plan answers admirably for cattle. It is simply the Scriptural plan of keeping the unclean apart from the clean. Infection no longer occurs and the disease dies out with those already affected. There seems no reason to doubt that the plan would give equally beneficent results with human beings, and many have urged its adoption. "But," it is said, "you cannot treat human beings as you treat cattle!" Are we then to treat them worse than we treat cattle? Are we to give security from infection to cattle and deny it to our children? Surely that is unthinkable. If segregation can free the race from this terrible scourge, some means must be found of making it so attractive to patients that they will willingly agree to it. Hitherto the difficulties have seemed insuperable, but now, I believe, they have been for the most part overcome. Many experiments have prepared the way, and all that is now necessary is to do on a large enough scale what has been done on a small. I have tried to sketch the outlines of a scheme on the necessary scale. Others no doubt will be able to point out defects and suggest improvements, and it will take the united wisdom of many minds to work it out fully.

### A National Unit.

I feel convinced that the provision of large colonies on garden-city lines, offering sufficient variety of work, of recreation, and of scenery, and the best possible hygienic conditions, is the only satisfactory way of dealing with our consumptive population—at least with those still able to work—and that, properly carried out, it will enable us to effect the speedy eradication of the disease. The scheme when fully developed ought to be a national one, as the county unit, unless in the case of

London, is too small to carry on a colony on the large scale which is necessary in order to secure the maximum of efficiency and economy. To safeguard the bulk of the population, geographical separation from the diseased is by far the easiest and most satisfactory arrangement. Some few of the healthy population, however, must of necessity remain in closer contact with the patients, and in order to safeguard them, and to keep the patients from reinfecting each other, special and somewhat elaborate arrangements are necessary, which are easy enough to carry out in colonies arranged for the purpose, but which it would be impossible to enforce in the general community. The possibility of thus preventing the spread of infection, even at close quarters, enables us to overcome one of the greatest difficulties in the way of a national segregation scheme—that of dealing with families in which the father or mother is affected, and in which complete separation from the rest of the family is undesirable. It also makes possible a certain amount of intercourse between patients and their healthy friends and associates who live outside the colonies.

### Palliating Segregation.

Under such a system patients fit for a fair day's work would still be able to support themselves and their families, and the inconveniences entailed by segregation would really be very trifling, while the advantages to both patients and public would be enormous. Surely it is better to face the small expense and inconvenience involved rather than to allow this loathsome disease to go on, generation after generation, sapping the life of the nation, carrying off not only our weaklings and ne'er-do-wells, but many of our bravest and best, and bringing about, not the survival of the fittest, as some fondly imagine, but a wholesale destruction of fit and unfit alike, and the maiming of many thousands whom it does not kill. The country should prepare at once to carry right through to a finish this campaign against consumption. I firmly believe complete victory is possible, and that at no distant date, and I appeal to one and all to help in the fight. I appeal to the Government and Local Authorities, for they must organise and combine the forces of the nation in the way most effective for action; to medical men and social reformers, for they must work out the plan of campaign and show the path to victory; to the rich, for I can imagine no more beneficent use for their riches; to the poor, for without their intelligent and cordial co-operation victory is impossible; to the healthy, that they may help the whole nation to enjoy the priceless blessings of health; and last, but not least, to the consumptives themselves, whose cheerful bravery under misfortune is proverbial, for though wounded soldiers in the fight they can yet render incomparable service to their country by willingly accepting the sacrifices segregation entails, in order that they may not pass on to others the disease which has marred their own lives. Even if their own wounds are mortal, they will then at least have this satisfaction, that when they die, this enemy of their country will die with them. Surely a grateful country will see that the sacrifices they are called upon to bear are made as light as possible.

I quite recognise that further preparation is needed before the nation can carry out such an ambitious scheme. When the Dreadnought type of unit was proposed for naval warfare a single Dreadnought had first to be built and tested, its capabilities ascertained, and its defects remedied. Then unit was added to unit, each embodying various improvements on its predecessor. In the campaign against tuberculosis we must proceed in the same way. A new fighting unit has been suggested

### A Test Experiment.

The plans look all right on paper, and there seems every reason to expect that the scheme will prove equal to what is required, but it has yet to be practically tested before we are justified in accepting the claims made for it. The immediate need is that funds should be raised and an organisation formed to start an experimental garden village for consumptives on a scale sufficient to afford a real and convincing test of the soundness of the scheme. A million pounds would be sufficient. Surely that is not too much to ask from a great and wealthy nation like this. The portion of our national income brought under review for income-tax purposes now exceeds a thousand million sterling, so that less than a farthing per pound of that income would be required if all contributed alike. It is absurd to shake our heads over the money difficulty or any other difficulty. The problem is pressing, for this is no leisurely preparation for war against a foe that we may have to fight some time or may never have to fight at all. We are now in the throes of the conflict. While we are marshalling our forces and making our plans, some 200 of our fellow citizens are perishing every day at the enemies' hands. Let us waste no time, then, in getting our new fighting unit ready for action. It will have to go into commission almost as soon as it is begun to be built. We cannot wait until it is finished. If it stands its tests satisfactorily, then we must go on adding larger and better units until our force is equal to its task, and speedy victory is assured.

### Note.

The scheme of colonies for the tuberculous advocated in this paper was first outlined by the author in a letter to the Editor of the *British Medical Journal*, which appeared under the heading "Tuberculosis Colonies" in the issue of September 21, 1912. Somewhat fuller outlines of the scheme appeared later in papers entitled "Tuberculosis: its Eradication," in the *Medical World* of August 7, 1913, and "Garden Cities for the Tuberculous," in the "Tuberculosis Year-Book and Sanatoria Annual, 1913." The present paper is being reprinted in pamphlet form, and will be published immediately by the Scientific Press, Limited. Price 3d.

